# Supplementary material for: On-Site and Visual Detection of the H5 Subtype Avian Influenza Virus Based on RT-RPA and CRISPR/Cas12a
Source: Viruses. 2024 May 10;16(5):753. doi: 10.3390/v16050753 (PMC11125590; doi:10.3390/v16050753)
Supplement: Supplementary file 1 [file viruses-16-00753-s001.zip › viruses-2988958-supplementary.pdf]

Table S1 The detection results of RT-RPA/CRISPR and RT-qPCR assay in clinical samples.

Number	Ct/Judge	RT-RPA/CRISPR/Judge	Virus isolation	Number	Ct/Judge	RT-RPA/CRISPR/Judge	Virus isolation
S1	26.346/P	262597/P	P	S42	34.600/N	11200/N	P
S2	35.629/N	6421/N	P	S43	29.907/P	69.04/N	P
S3	29.778/P	1432471/P	P	S44	32.101/P	30621/P	P
S4	27.751/P	466241/P	P	S45	35.455/N	5547/N	P
S5	34.693/P	105884/P	P	S46	23.372/P	906606/P	P
S6	31.793/P	61936/P	P	S47	24.171/P	965443/P	P
S7	10.859/P	619727/P	P	S48	23.864/P	696776/P	P
S8	25.998/P	448527/P	P	S49	36.242/N	2159/N	P
S9	20.677/P	893843/P	P	S50	17.096/P	849464/P	P
S10	30.652/P	80363/P	P	S51	28.375/P	7792/N	P
S11	28.063/P	929154/P	P	S52	26.453/P	904615/P	P
S12	31.326/P	50958/P	P	S53	29.150/P	9956/N	P
S13	20.782/P	723319/P	P	S54	18.267/P	942682/P	P
S14	35.860/N	7460/N	P	S55	21.280/P	1013/N	P
S15	22.925/P	619727/P	P	S56	19.452/P	702150/P	P
S16	21.778/P	788743/P	P	S57	-/N	3678/N	P
S17	23.189/P	1300663/P	P	S58	36.064/N	1150/N	N
S18	31.710/P	61963/P	P	S59	35.146/N	885/N	N
S19	31.849/P	22875/P	P	S60	36.034/N	322/N	N
S20	35.383/N	33720/P	P	S61	35.583/N	2406/N	N
S21	28.800/P	170584/P	P	S62	37.171/N	1177/N	N
S22	18.739/P	803524/P	P	S63	35.858/N	1093/N	N

Table S1（continue）

Number	Ct/Judge	RT-RPA/CRISPR/Judge	Virus isolation	Number	Ct/Judge	RT-RPA/CRISPR/Judge	Virus isolation
S23	34.050/N	43637/P	P	S64	35.114/N	4843/N	N
S24	18.765/P	1280066/P	P	S65	-/N	778/N	N
S25	24.864/P	737056/P	P	S66	17.275/P	761806/P	N
S26	26.965/P	556659/P	P	S67	37.624/N	1347/N	N
S27	32.239/P	63254/P	P	S68	35.176/N	1951/N	N
S28	23.767/P	629848/P	P	S69	35.090/N	1177/N	N
S29	18.753/P	890329/P	P	S70	37.605/N	2927/N	N
S30	23.341/P	1458743/P	P	S71	37.792/N	836/N	N
S31	30.129/P	40308/P	P	S72	37.138/N	437/N	N
S32	31.267/P	33720/P	P	S73	36.712/N	2718/N	N
S33	31.609/P	42936/P	P	S74	35.362/N	1831/N	N
S34	22.473/P	628337/P	P	S75	36.219/N	1023/N	N
S35	23.730/P	1519676/P	P	S76	-/N	2257/N	N
S36	18.993/P	1163838/P	P	S77	35.254/N	1093/N	N
S37	28.758/P	771067/P	P	S78	37.255N	2927/N	N
S38	35.875/N	403011/P	P	S79	35.713N	2818/N	N
S39	36.938/N	942682/P	P	S80	21.101/P	156403/P	N
S40	35.677/N	929605/P	P	S81	29.848/P	28517/P	N
S41	35.094/N	7036/N	P				

Note: “P”: Positive；“N”: Negative；“S”: Sample；S1-S57 are positive samples；S58-S81 are negative samples.
